# Patterns of Energy Drink Use, Risk Perception, and Regulatory Attitudes in the Adult Polish Population: Results of a Cross-Sectional Survey

**DOI:** 10.3390/nu17091458

**Published:** 2025-04-26

**Authors:** Paulina Mularczyk-Tomczewska, Aleksandra Lewandowska, Agnieszka Kamińska, Małgorzata Gałecka, Paweł A. Atroszko, Tomasz Baran, Tytus Koweszko, Andrzej Silczuk

**Affiliations:** 1 Department of Public Health, Faculty of Health Sciences, Medical University of Warsaw, 02-091 Warsaw, Poland; 2 J. Babinski Specialist Psychiatric Health Care Team, Psychiatric Ward of Children, 91-229 Lodz, Poland; aleksandra_lewandowska@poczta.onet.pl; 3 Ophthalmology Department, Faculty of Medicine, Collegium Medicum, Cardinal Stefan Wyszyński University in Warsaw, 01-938 Warsaw, Poland; agnieszka.kaminska73@wp.pl; 4 Department of Psychotherapy, Faculty of Medicine, Medical University of Lodz, 91-229 Lodz, Poland; malgorzata.galecka@umed.lodz.pl; 5 Department of Psychometrics and Statistics, Social Sciences Faculty, Institute of Psychology, University of Gdansk, 80-309 Gdansk, Poland; pawel.atroszko@ug.edu.pl; 6 Faculty of Psychology, University of Warsaw, 00-183 Warsaw, Poland; tomasz.baran@panelariadna.pl; 7 Department of Community Psychiatry, Faculty of Health Sciences, Medical University of Warsaw, 02-091 Warsaw, Poland; tytus.koweszko@wum.edu.pl (T.K.); andrzej.silczuk@wum.edu.pl (A.S.)

**Keywords:** energy drinks, caffeine and taurine, adolescent health, risk perception, sociodemographic factors, educational campaigns, public health protection

## Abstract

**Background:** From 1 January 2024, Poland legally banned the sale of energy drinks (EDs) containing caffeine and taurine to minors under 18. EDs are rich in stimulants, making them particularly popular among adolescents and young adults seeking energy boosts. Their consumption is associated with adverse health effects and engagement in risky behaviors. This descriptive snapshot study explores energy drink consumption, motivations, and risk perception among Polish adults. **Methods:** A cross-sectional, nationwide survey (N = 1121) was conducted over a three-day period using the computer-assisted web interviewing (CAWI) method. The sample was representative of the adult Polish population in terms of gender, age, and geographical location. Data collection occurred 14 months after the implementation of legal restrictions on ED sales to minors. **Results:** Among 1121 adults (54.3% female), 15.1% reported weekly ED consumption, 9.7% monthly, 13.3% less than monthly, and 62.0% never. Younger age (*p* < 0.05), male gender (aOR = 1.63 [1.25–2.13]; *p* < 0.001), and active occupational status (aOR = 1.63 [1.19–2.24]; *p* = 0.002) were significantly associated with ED use. Overall, 83.0% of respondents perceived EDs as harmful, and 87.2% supported the sales ban to minors, although only 45.6% considered the ban effective. Additionally, 31.8% had observed minors consuming EDs in the past 30 days. Sociodemographic characteristics were associated with risk perception and support for regulation: women (aOR = 1.50), individuals with higher education (aOR = 1.44), and those with good financial status (aOR = 2.35) were more likely to perceive EDs as harmful and endorse regulatory measures. **Conclusions:** Educational interventions should prioritize young adults, particularly males, who constitute the primary consumers. There is also a need to enhance enforcement mechanisms to limit minors’ access to energy drinks and curb their marketing and availability, thereby improving public health protection.

## 1. Introduction

Energy drinks (EDs), first introduced in Asia in the 1960s, have rapidly gained popularity worldwide [1]. Over the years, their consumption has steadily increased, becoming a global trend [2]. Since their introduction to the European Market in 1987, the consumption of EDs has grown significantly. By 2020, the global ED market was valued at USD 45.80 billion, with projections indicating an annual growth rate of 8.2% and reaching USD 108.40 billion by 2031 [3]. As their popularity continues to rise, concerns about the potential health risks associated with ED consumption have also grown.

EDs are non-alcoholic beverages containing high amounts of caffeine (≥150 mg/L) and sugar, along with other stimulants such as taurine, ginseng, and guarana [4]. In Poland, under Article 12l of the Public Health Act of 11 September 2015, an ED is defined as a beverage that contains added caffeine (CAF) or taurine (TA) and is categorized within class 10.89 or division 11 of the Polish Classification of Goods and Services [5]. A product meets this classification if its caffeine content exceeds 150 mg/L or if it includes taurine, except when these substances are naturally occurring in the drink [6]. EDs are beverages designed to enhance mental alertness and physical performance by stimulating the nervous system [1]. They are widely promoted for their claimed benefits, including increased energy and alertness, reduced fatigue, enhanced physical performance, boosted metabolic activity, and various other physiological, cognitive, and performance improvements [7]. However, these claimed benefits come with associated risks, particularly regarding their long-term impact on health.

Although certain cardiovascular side effects, like high blood pressure, are widely recognized, EDs continue to be highly popular, particularly among teenagers [8]. The intake of EDs has been associated with poor dietary habits, weight gain, and mental health issues among adolescents [9,10,11,12]. This growing popularity among minors has raised concerns, with studies showing a significant increase in emergency department admissions linked to ED consumption [13]. Further studies indicate that energy drinks can have harmful effects on various organs, leading to mild symptoms like anxiety, gastrointestinal issues, dehydration, nervous agitation, and an increased heart rate. In severe cases, they can cause rhabdomyolysis, acute kidney injury, ventricular fibrillation, seizures, manic episodes, and stroke [14,15,16,17]. There have also been documented fatalities related to ED consumption [18,19,20]. Given the growing evidence of health risks, there has been increasing support for stronger regulatory measures to restrict the sale of EDs to vulnerable groups, particularly minors.

Despite evidence of potential health risks, EDs remain widely consumed by young people [21]. The NOMISMA-ARETÉ Consortium for the European Food Safety Authority (EFSA) reported that ED consumption was most prevalent among adolescents (68%), while lower rates were observed in adults (30%) and children (18%) [22]. Adolescents often try EDs before the age of 12 [23], and teenage boys tend to consume them more frequently than girls. Frequent consumers of EDs also tend to have higher intake of soft drinks, greater alcohol consumption, more screen time, and later bedtimes [24].

The long-term effects of regular ED consumption remain insufficiently studied, particularly regarding potential risks for adolescents. Despite this, growing scientific discussion on the harmful consequences of these beverages is raising awareness of their potential dangers [25,26,27]. Due to the still limited knowledge about EDs, it is essential to implement legal regulations, including age restrictions, greater ingredient transparency, and clear labeling of adverse effects [28].

The growing popularity of EDs, coupled with a lack of proper regulations, has led to calls for banning their sale to minors [29]. As concerns about the safety of EDs grow, many countries have begun to implement regulations to protect vulnerable groups, such as minors. Countries such as Lithuania, Latvia, Turkey, and Sweden have implemented regulations prohibiting the sale of EDs to individuals under 18 [30,31,32,33]. In Spain, the Agency for Food Safety and Nutrition (AESAN) recommends that minors, as well as pregnant and breastfeeding women, avoid consuming EDs [34]. In the United States, while no formal age restrictions exist, various organizations have expressed significant concerns regarding the potential health risks posed by energy drink consumption among children, adolescents, and young adults [35].

In response to growing concerns about ED consumption among minors, Poland has introduced legal restrictions aimed at reducing their intake. Starting from 1 January 2024, under Article 12m of the Public Health Act, the sale of beverages containing CAF or TA to individuals under the age of 18 is prohibited. Additionally, this ban extends to sales in schools, as outlined in the Education Act, as well as in vending machines. If there is any doubt about a buyer’s age, sellers are authorized to request identification for verification [5].

The objectives of this study, conducted on adults in Poland, were to assess (1) the frequency of ED consumption and reasons for consumption in adults, (2) opinions on the health risks of EDs and their impact on health, and (3) support for a ban on the sale of EDs to minors and the need for additional restrictions, such as advertising limitations.

## 2. Materials and Methods

### 2.1. Study Design and Population

This cross-sectional survey was conducted on a representative nationwide Polish sample (N = 1121) between 28 February 2025 and 3 March 2025. The computer-assisted web interview (CAWI) technique was used. All the participants were recruited using the Ariadna Polish online panel [36], which has over 110,000 active panel members aged 15 and over, provides representativeness for the Polish population, and is actively updated to maintain representativeness for the Polish population. The survey method was the use of the computer-assisted web interview (CAWI). The process of selecting the research sample was conducted in two phases. In the first phase, the population was divided into mutually exclusive subgroups based on key demographic factors such as gender, age, place of residence, region, and education level. In the next phase, respondent recruitment was carried out based on demographic data provided by the Central Statistical Office in Poland. The Central Statistical Office in Poland, officially known as Statistics Poland (GUS), is the main government agency responsible for collecting and publishing official statistics in the country. It gathers data related to the economy, population, and society at both the national and local levels [37].

Participation in the study was voluntary and anonymous. Informed consent was collected from all the participants. The study protocol was reviewed and approved by the Ethical Review Board at the Medical University of Warsaw, Poland (decision number AKBE/37/2025).

### 2.2. Questionnaire and Study Measures

The study questionnaire was developed based on a literature review and included eight questions on public attitudes and behaviors related to energy drink consumption (Table A2, Appendix A). Public attitudes towards the health effects of energy drink consumption, support for a ban on the sale of energy drinks to minors, the perception of such a ban as an effective measure, and support for further regulation of energy drinks (e.g., advertising ban) were measured using a 5-point Likert scale. Respondents who answered “definitely yes” or “mostly yes” were considered to be expressing positive views related to the measured attitude.

To assess ED consumption, respondents were asked whether they had ever consumed energy drinks, whether they had consumed them in the past 30 days, and what their reasons for consumption were. Awareness of energy drinks was evaluated by asking whether respondents believed energy drinks were harmful to health and whether, in their opinion, consuming them could cause specific health issues such as increased blood pressure, irritability, anxiety, nausea, vomiting, dizziness, sleep disturbances, or impaired motor coordination. The third section of the questionnaire focused on respondents’ attitudes toward the recently introduced ban on selling energy drinks to individuals under 18. Participants were asked whether they believed the ban had effectively reduced the availability of EDs to minors, whether they had witnessed a person under 18 consuming an energy drink in the past 30 days, and whether they thought additional restrictions should be introduced, such as advertising limitations.

Questions related to sociodemographic data included the following: gender (male/female), age (years), place of residence (rural; city below 20,000 residents; city from 20,000 to 99,999 residents; city from 100,000 to 499,999 residents; city above 500,000 residents); educational level (primary, secondary, or higher), occupational status (employed under an employment contract, employed under a contract of mandate, employed under a contract for specific work, self-employed, unemployed, retiree or pensioner, pupil or student, homemaker, or other), and household composition of respondents (whether they lived with children under the age of 18).

### 2.3. Statistical Analysis

Data were analyzed using IBM SPSS Statistics version 29.0 (IBM Corp., Armonk, NY, USA). Descriptive statistics, including frequencies and proportions, were used to summarize the distribution of categorical variables. Associations between categorical variables were assessed using cross-tabulations and Pearson’s chi-square test. To identify factors associated with energy drink (ED) consumption and public attitudes toward EDs, logistic regression analyses were conducted.

Two separate logistic regression models were developed to examine the determinants of ED consumption:

Model 1 included the full study sample (N = 1121) and compared individuals who reported any level of ED consumption (including those consuming less than once per month) with those who reported no consumption (non-consumers).

Model 2 was restricted to participants who reported ED consumption (n = 426) and was used to identify factors associated with more frequent or specific patterns of ED use within this subgroup.

In addition, two further logistic regression models were constructed to investigate: factors associated with the perception of EDs as harmful to health, and determinants of support for legislation banning the sale of EDs to minors. In the bivariate analysis phase, eight independent variables were evaluated individually for their association with the respective dependent variables. Only variables that reached statistical significance (*p* < 0.05) in the bivariate analyses were included in the final multivariable logistic regression models.

The strength of associations was expressed as odds ratios (ORs) with corresponding 95% confidence intervals (95% CI). Statistical significance was set at *p* < 0.05 for all analyses.

## 3. Results

Survey questionnaires were collected from 1121 adults aged 18–86 years, 54.3% of whom were females. The age distribution was as follows: 14.2% were 18–29 years, 19.4% were 30–39 years, 19.7% were 40–49 years, 18.6% were 50–59 years, 17.8% were 60–69 years, and 10.3% were 70 years or older. Higher education was reported by 45.0% of participants. The majority (55.7%) were married. Employment status showed that 60.6% were actively working (employed or self-employed), while 39.4% were unemployed, students, or pensioners. Financial self-assessment indicated that 48.3% considered their household status as good, 37.6% as moderate, and 14.2% as poor. A total of 37.3% resided in rural areas, while 62.7% lived in urban settings. Additionally, 27.4% of respondents had children under 18 years of age. Detailed characteristics of the study population are presented in Table A1 (Appendix A).

### 3.1. Public Perception of Energy Drinks

Among respondents, 15.1% consumed energy drinks at least once a week, 9.7% at least once a month, 13.3% less than once a month, and 62% did not drink energy drinks at all. Males and younger adults were more likely (*p* < 0.05) to consume energy drinks. Most of the respondents (83%) declared that energy drinks are harmful to health. Support for a ban on ED sales to minors was declared by 87.2% of respondents. However, less than half of respondents (45.6%) believed that the ban on energy drink sales to minors was effective. One-third of respondents had seen minors consuming EDs in the previous 30 days. Attitudes towards ED consumption and law regulation differed by sociodemographic variables (*p* < 0.05) (see Table A2, Appendix A).

### 3.2. Sociodemographic Differences in the Frequency of Using EDs

In multivariable logistic regression, male gender was significantly associated with more frequent ED consumption (*p* = 0.002), with a higher percentage of men consuming EDs at least once a week compared to women (18.0% vs. 12.6%). Younger age was also a key factor (*p* < 0.001), with the highest frequency of ED consumption observed in the 18–29 age group (28.9% reported drinking at least once a week). The frequency of consumption decreased with age, with only 0.9% of individuals aged 70+ consuming energy drinks at least once a week. Marital status was significantly associated with consumption (*p* < 0.001), with unmarried individuals more likely to consume energy drinks frequently. Additionally, individuals with children under 18 years were significantly more likely to consume energy drinks at least once a week (23.1%) compared to those without children (12.0%) (*p* < 0.001).

Occupational status was also an important factor (*p* < 0.001), with actively employed individuals consuming energy drinks more frequently (20.2% at least once a week) compared to those who were unemployed, students, or retirees (7.2%). Self-declared financial status was not significantly associated with energy drink consumption (*p* = 0.6), indicating that income perception did not strongly influence drinking habits (see Table A3, Appendix A).

### 3.3. Public Perception of EDs and Sociodemographic Variables

In multivariable logistic regression, the female gender was significantly associated with the perception of energy drinks as harmful to health (85.6% vs. 80.1% in males, *p* = 0.02) and with support for a ban on the sale of energy drinks to minors (89.5% vs. 84.4%, *p* = 0.01). Women were also significantly more likely to support further regulations, such as advertising restrictions (74.4% vs. 61.9%, *p* < 0.001).

Age was a key determinant in public perception. Older individuals were more likely to support the ban on sales to minors (*p* = 0.001), with the highest agreement observed in those aged 70+ (93.0%). They were also significantly more likely to support additional restrictions (*p* < 0.001), with 81.7% of individuals aged 70+ in favor. Younger individuals (18–29 years) were more likely to have observed minors consuming energy drinks in the past 30 days (45.9%, *p* < 0.001).

Higher education was significantly associated with perceiving EDs as harmful (86.5% vs. 80.2%, *p* = 0.01) and supporting a ban on sales to minors (89.9% vs. 84.9%, *p* = 0.01). However, it was not significantly linked to the perception that the ban effectively limited availability (*p* = 0.2). Married individuals were more likely to perceive energy drinks as harmful (85.1% vs. 80.5%, *p* = 0.04) and to support the ban (89.7% vs. 83.9%, *p* = 0.004), but no significant difference was observed in their perception of the ban’s effectiveness (*p* = 0.01). Individuals with a good self-declared financial status were significantly more likely to support the ban on sales to minors (92.1% vs. 83.6% in those with poor financial conditions, *p* < 0.001) and additional restrictions (70.6% vs. 64.8%, *p* = 0.09). However, those with moderate financial status were less likely to perceive energy drinks as harmful (77.2% vs. 87.1%, *p* < 0.001). Finally, individuals with children under 18 were more likely to have observed minors consuming EDs in the previous 30 days (38.1% vs. 29.5%, *p* = 0.006) (Table A4, Appendix A).

### 3.4. Factors Associated with the Frequency of ED Consumption

In multivariable logistic regression models, younger age (*p* < 0.05), male gender (aOR = 1.63 [1.25–2.13]; *p* < 0.001), and active occupational status (aOR = 1.63 [1.19–2.24]; *p* = 0.002) were significantly associated with energy drink consumption (Table A5). Among those who consumed EDs, age of 18–29 years (aOR = 9.33 [1.15–75.97]; *p* = 0.04) was the only sociodemographic variable associated with energy drink consumption at least once a week (Table A5, Appendix A).

### 3.5. Factors Associated with the Public Perception of EDs

In multivariable logistic regression, female gender (aOR = 1.50 [1.09–2.06]; *p* = 0.01) and having higher education (aOR = 1.44 [1.03–2.00]; *p* = 0.03) were significantly associated with public perception of EDs as products harmful to health (Table A6). Individuals with moderate self-declared household financial status were less likely to declare that energy drinks are harmful to health (aOR = 0.59 [0.36–0.96; *p* = 0.04] compared to those with bad economic conditions. Female gender (aOR = 1.68 [1.16–2.41]; *p* = 0.005], age 40 years and over (*p* < 0.05), and good self-declared household financial status (aOR = 2.35 [1.37–4.03]; *p* = 0.002) were significantly associated with the support declaration for the ban on the sale of EDs to minors (Table A6, Appendix A).

## 4. Discussion

The study found that a portion of adults consume energy drinks, with younger age, male gender, and active occupational status linked to higher consumption. Most participants viewed energy drinks as harmful and supported a ban on sales to minors, though many doubted its effectiveness. A significant number had also observed minors consuming energy drinks. Women, those with higher education, and those with better financial status were more likely to perceive energy drinks as harmful and support the ban.

Although a legal ban on the sale of EDs to minors was implemented in Poland in 2024, the study findings suggest that perceived access among minors may still be an issue, as some respondents reported witnessing individuals they believed to be under 18 consuming energy drinks. However, as this observation is based on self-report and subjective judgment, it cannot be conclusively determined whether those individuals were in fact minors. In particular, 31.8% of respondents reported having witnessed minors consuming EDs in the previous 30 days. This suggests that while regulatory measures are in place, enforcement challenges persist. Similar concerns have been raised at the European level. A 2024 policy analysis emphasized the necessity of stricter enforcement mechanisms to prevent underage access, highlighting that mere legislative action is insufficient without robust monitoring and retailer compliance measures [9].

Public awareness of the health risks associated with ED consumption is relatively high in Poland, with 83% of respondents perceiving them as harmful. This aligns with a growing body of evidence documenting the adverse cardiovascular, neurological, and metabolic effects of EDs. Furthermore, studies have shown that mixing EDs with alcohol, a common behavior among adolescents, can severely impair brain function, particularly in regions responsible for learning and memory, such as the hippocampus [38].

ED consumption has also been linked to behavioral and mental health concerns. A systematic review in 2021 found that frequent consumers of EDs, particularly adolescents, exhibit a higher prevalence of anxiety, depression, and impulsivity-related behaviors [39]. Similarly, a 2022 review identified strong correlations between ED intake and increased engagement in risky behaviors such as substance use, excessive screen time, and poor dietary choices [27]. These findings align with the present study’s results, which indicate that younger males are the most frequent consumers of EDs, a pattern consistent with broader international trends [29].

Although public support for regulatory measures is strong—87.2% of respondents favor banning ED sales to minors, and 68.7% support additional restrictions, such as advertising bans—only 45.6% believe the current ban has effectively reduced access. This discrepancy underscores the limitations of legislative measures that are not coupled with enforcement mechanisms and public education campaigns. Prior research has emphasized the need for multifaceted approaches, including age verification measures, retailer accountability, and awareness campaigns to improve policy effectiveness [9,13].

From a market perspective, the global energy drink industry continues to expand, with projections estimating its value to exceed USD 100 billion by 2031 [39]. This growing market presence presents additional challenges for regulation as ED manufacturers target younger demographics through aggressive marketing strategies. A 2023 review highlighted the urgent need for policies addressing ED marketing practices, given their role in shaping consumer behavior and increasing appeal among minors [11]. Energy drink consumption has become an increasing public health concern [40].

Limitations of the study: This cross-sectional survey has several limitations. The data relied on self-reports, which may be subject to recall bias. Data regarding the use of EDs, as well as witnessing its use in minors, were self-reported and were not verified against medical records. This study does not aim to evaluate the effectiveness of the 2024 policy banning ED sales to minors. As a cross-sectional snapshot without pre–post comparison or control groups, it cannot determine causal relationships or policy impact. Instead, it provides a timely overview of consumption patterns, public attitudes, and perceived enforcement issues, which may inform future evaluation frameworks. Finally, as the study used the CAWI method, it only included respondents with internet access, potentially limiting the diversity of the sample.

### Policy Implications and Strategic Recommendations

The sociodemographic differentiation observed in this study suggests a need for stratified and evidence-based public health interventions. Given the disproportionate prevalence of energy drink (ED) consumption among young adults (particularly those aged 18–29), males, and economically active individuals, targeted regulatory policies are warranted. While public support for restrictions on ED sales to minors is high, the perception of enforcement effectiveness remains limited. This implies an implementation gap that could be addressed through enhanced compliance monitoring and retailer accountability mechanisms (e.g., mandatory ID verification, fiscal penalties for non-compliance). Moreover, integrating ED-related health risk education into school curricula and workplace wellness programs may increase the preventive impact. Considering that higher education and female gender are positively associated with risk perception and support for regulation, public awareness campaigns could benefit from tailored messaging that resonates with males and younger audiences. Finally, in light of the observed discrepancy between support for bans and the continued visibility of minors consuming EDs, longitudinal evaluations of legislative impact and accessibility should be prioritized in future research agendas.

## 5. Conclusions

Despite the ban on the sale of energy drinks (EDs) to minors introduced in Poland on 1 January 2024, the findings from this CAWI-based survey suggest that these products may still be accessible to underage individuals. However, due to the nature of the data collection method, which relies on self-reported responses, these observations should be interpreted with caution. Energy drink consumption remains highest among young males, with a notable decline in older age groups. While most respondents perceive EDs as harmful, concerns are more pronounced among women and individuals with higher education. Furthermore, women, older adults, and those with higher education are more likely to support further restrictions on EDs. These results emphasize the need for more robust research methodologies, including observational and longitudinal studies, to better assess the true impact of regulatory measures and the ongoing accessibility of energy drinks to minors. Future research should also address potential biases in self-reported data and further explore the factors influencing public perception and behavior related to ED consumption.

## Data Availability

The data supporting the results of this study will be available upon request from interested researchers. If the data cannot be made publicly available in a trusted repository, the reason for this will be specified in the Data Availability Statement. Further information and materials necessary for the reproduction of the experiment can be obtained by contacting the authors.

## Abbreviations

The following abbreviations are used in this manuscript:

EDs	Energy drinks
OR	Odds ratio
CI	Confidence intervals
CAWI	Computer-assisted web interview

## Appendix A

**Table A1:** Characteristics of the study population (n = 1121).

Variable	n	%
**Gender**		
Female	609	54.3
Male	512	45.7
**Age [years]**		
18–29	159	14.2
30–39	218	19.4
40–49	221	19.7
50–59	209	18.6
60–69	199	17.8
>70	115	10.3
**Higher education**		
Yes	504	45.0
No	617	55.0
**Married**		
Yes	624	55.7
No	497	44.3
**Place of residence**		
Rural area	418	37.3
City of <20,000 residents	143	12.8
City of 20,000–99,999 residents	226	20.2
City of 100,000–499,999 residents	197	17.6
City of ≥500,000 residents	137	12.2
**Having children under 18 years**		
Yes	307	27.4
No	814	72.6
**Occupational status**		
Active (employed or self-employed)	679	60.6
Passive (unemployed, student, or pensioner)	442	39.4
**Self-declared household financial status**		
Good	541	48.3
Moderate	421	37.6
Bad	159	14.2

**Table A2:** Public perception of energy drinks among adults in Poland (n = 1121).

Variable	n	%
**How often do you drink energy drinks?**		
Every day or almost every day	41	3.7
2–3 times a week	65	5.8
Once a week	63	5.6
2–3 times a month	67	6.0
Once a month	41	3.7
Less than once a month	149	13.3
Never	695	62.0
**What is the main reason you drink energy drinks? (n = 426) [multiple-choice question]**		
To stimulate and increase energy	193	45.3
To improve concentration and mental performance	111	26.1
To improve physical performance	80	18.8
To improve mood	70	16.4
Due to the taste of the drink	127	29.8
Out of habit, I feel the need to drink	41	9.6
Under the influence of environmental pressure	25	5.9
In combination with alcohol	36	8.5
As an alternative to coffee	94	22.1
Other	9	2.1
**Do you think that energy drinks are harmful to health?**		
Definitely yes	527	47.0
Mostly yes	404	36.0
Mostly no	54	4.8
Definitely no	15	1.3
I do not know/difficult to tell	121	10.8
**What health effects do you think energy drinks can cause? [multiple-choice question]**		
Increased blood pressure	760	67.8
Irritability, anxiety	658	58.7
Nausea, vomiting	294	26.2
Dizziness	403	36.0
Sleep problems	719	64.1
Impaired motor coordination	300	26.8
I don’t know	23	21
**Do you support a ban on the sale of energy drinks to minors (under 18 years of age)?**		
Definitely yes	708	63.2
Mostly yes	269	24.0
Mostly no	39	3.5
Definitely no	33	2.9
I do not know/difficult to tell	72	6.4
**Do you think that the introduction of the ban on the sale of energy drinks over the past year (from 1 January 2024) has effectively limited their availability to minors?**		
Definitely yes	159	14.2
Mostly yes	352	31.4
Mostly no	301	26.9
Definitely no	92	8.2
I do not know/difficult to tell	217	19.4
**Have you seen anyone under the age of 18 consume an energy drink in the last 30 days?**		
Yes	357	31.8
No	764	68.2
**Do you think there should be additional restrictions on energy drinks, such as limiting advertising?**		
Definitely yes	383	34.2
Mostly yes	387	34.5
Mostly no	124	11.1
Definitely no	60	5.4
I do not know/difficult to tell	167	14.9

**Table A3:** Sociodemographic differences in the frequency of using EDs (n = 1121).

	Frequency of Drinking Energy Drinks in the Last 30 Days				
Variable	At Least Once a Week (n = 169)	1–3 Times a Month (n = 108)	Less Than Once a Month (n = 149)	Never (n = 695)	*p*
**Gender**					
Female	77 (12.6)	52 (8.5)	72 (11.8)	408 (67.0)	0.002
Male	92 (18.0)	56 (10.9)	77 (15.0)	287 (56.1)	
**Age [years]**					
18–29	46 (28.9)	24 (15.1)	24 (15.1)	65 (40.9)	<0.001
30–39	48 (22.0)	31 (14.2)	33 (15.1)	106 (48.6)	
40–49	47 (21.3)	23 (10.4)	39 (17.6)	112 (50.7)	
50–59	18 (8.6)	22 (10.5)	23 (11.0)	146 (69.9)	
60–69	9 (4.5)	5 (2.5)	20 (10.1)	165 (82.9)	
>70	1 (0.9)	3 (2.6)	10 (8.7)	101 (87.8)	
**Higher education**					
Yes	71 (14.1)	46 (9.1)	77 (15.3)	310 (61.5)	0.3
No	98 (15.9)	62 (10.0)	72 (11.7)	385 (62.4)	
**Married**					
Yes	75 (12.0)	60 (9.6)	72 (11.5)	417 (66.8)	<0.001
No	94 (18.9)	48 (9.7)	77 (15.5)	278 (55.9)	
**Place of residence**					
Rural area	51 (12.2)	42 (10.0)	52 (12.4)	273 (65.3)	0.5
City of <20,000 residents	19 (13.3)	19 (12.6)	15 (10.5)	91 (63.6)	
City of 20,000–99,999 residents	42 (18.6)	20 (8.8)	34 (15.0)	130 (57.5)	
City of 100,000–499,999 residents	35 (17.8)	18 (9.1)	26 (13.2)	118 (59.9)	
City of ≥500,000 residents	22 (16.1)	10 (7.3)	22 (16.1)	83 (60.6)	
**Having children under 18 years**					
Yes	71 (23.1)	41 (13.4)	38 (12.4)	157 (51.1)	<0.001
No	98 (12.0)	67 (8.2)	111 (13.6)	538 (66.1)	
**Occupational status**					
Active (employed or self-employed)	137 (20.2)	83 (12.2)	100 (14.7)	359 (52.9)	<0.001
Passive (unemployed, student, or pensioner)	32 (7.2)	25 (5.7)	49 (11.1)	336 (76.0)	
**Self-declared household financial status**					
Good	78 (14.4)	54 (10.0)	77 (14.2)	332 (61.4)	0.6
Moderate	64 (15.2)	45 (10.7)	52 (12.4)	260 (61.8)	
Bad	27 (17.0)	9 (5.7)	20 (12.6)	103 (64.8)	

**Table A4:** Public perception of EDs by sociodemographic variables (n = 1121).

Variable	Energy Drinks Are Harmful to Health		Support a Ban on the Sale of Energy Drinks to Minors		Perception of the Ban on Selling Energy Drinks to Minors as an Effective Measure		Observation of Minors Drinking Energy Drinks in the Last 30 Days		Support for Further Regulation of Energy Drinks, e.g., Advertising Ban	
	n (%)	*p*	n (%)	*p*	n (%)	*p*	n (%)	*p*	n (%)	*p*
**Gender**										
Female	521 (85.6)	0.02	545 (89.5)	0.01	279 (45.8)	0.9	185 (30.4)	0.3	453 (74.4)	<0.001
Male	410 (80.1)		432 (84.4)		232 (45.3)		172 (33.6)		317 (61.9)	
**Age [years]**										
18–29	127 (79.9)	0.6	125 (78.6)	0.001	63 (39.6)	0.6	73 (45.9)	<0.001	88 (55.3)	<0.001
30–39	183 (83.9)		183 (83.9)		99 (45.4)		65 (29.8)		138 (63.3)	
40–49	179 (81.0)		195 (88.2)		108 (48.9)		74 (33.5)		143 (64.7)	
50–59	181 (86.6)		191 (91.4)		100 (47.8)		74 (35.4)		159 (76.1)	
60–69	164 (82.4)		176 (88.4)		89 (44.7)		46 (23.1)		148 (74.4)	
70	97 (84.3)		107 (93.0)		52 (45.2)		25 (21.7)		94 (81.7)	
**Higher education**										
Yes	436 (86.5)	0.01	453 (89.9)	0.01	218 (43.3)	0.2	167 (33.1)	0.4	361 (71.6)	0.06
No	495 (80.2)		524 (84.9)		293 (47.5)		190 (30.8)		409 (66.3)	
**Married**										
Yes	531 (85.1)	0.04	560 (89.7)	0.004	305 (48.9)	0.01	201 (32.2)	0.8	448 (71.8)	0.01
No	400 (80.5)		417 (83.9)		206 (41.4)		156 (31.4)		322 (64.8)	
**Place of residence**										
Rural area	348 (83.3)	0.9	359 (85.9)	0.7	197 (47.1)	0.8	120 (28.7)	0.03	281 (67.2)	0.2
City of <20,000 residents	118 (82.5)		128 (89.5)		61 (42.7)		48 (33.6)		107 (74.8)	
City of 20,000–99,999 residents	189 (83.6)		195 (86.3)		100 (44.2)		87 (38.5)		149 (65.9)	
City of 100,000–499,999 residents	160 (81.2)		172 (87.3)		94 (47.7)		52 (26.4)		132 (67.0)	
City of ≥500,000 residents	116 (84.7)		123 (89.8)		59 (43.1)		50 (36.5)		101 (73.7)	
**Having children under 18 years**										
Yes	257 (83.7)	0.7	274 (89.3)	0.2	163 (53.1)	0.002	117 (38.1)	0.006	216 (70.4)	0.5
No	674 (82.8)		703 (86.4)		348 (42.8)		240 (29.5)		554 (68.1)	
**Occupational status**										
Active (employed or self-employed)	569 (83.8)	0.4	596 (87.8)	0.4	330 (48.6)	0.01	244 (35.9)		459 (67.6)	0.3
Passive (unemployed, student, or pensioner)	362 (81.9)		381 (86.2)		181 (41.0)		113 (25.6)	<0.001	311 (70.4)	
**Self-declared household financial status**										
Good	471 (87.1)	<0.001	498 (92.1)	<0.001	256 (47.3)	0.5	181 (33.5)	0.002	382 (70.6)	0.09
Moderate	325 (77.2)		346 (82.2)		183 (43.5)		111 (26.4)		273 (64.8)	
Bad	135 (84.9)		133 (83.6)		72 (45.3)		65 (40.9)		115 (72.3)	

**Table A5:** Factors associated with the frequency of ED consumption.

Variable	Energy Drinks Consumption (n = 1121)				Consumption of Energy Drinks at Least Once a Week, Among Those Who Drink Energy Drinks (n = 426)			
	Bivariable Logistic Regression		Multivariable Logistic Regression		Bivariable Logistic Regression		Multivariable Logistic Regression	
	OR (95%CI)	*p*	aOR (95%CI)	*p*	OR (95%CI)	*p*	aOR (95%CI)	*p*
**Female**	Ref.		Ref		0.90 (0.61–1.33)	0.6		
**Male**	1.59 (1.25–2.03)	<0.001	1.63 (1.25–2.13)	<0.001	Ref.			
**Age [years]**								
18–29	10.43 (5.49–19.83)	<0.001	7.53 (3.76–15.09)	<0.001	12.46 (1.57–99.10)	0.02	9.33 (1.15–75.97)	0.04
30–39	7.62 (4.11–14.15)	<0.001	4.99 (2.49–9.98)	<0.001	9.75 (1.23–77.12)	0.03	6.12 (0.74–50.76)	0.1
40–49	7.02 (3.78–13.03)	<0.001	4.41 (2.20–8.84)	<0.001	9.86 (1.26–78.02)	0.03	6.31 (0.76–52.03)	0.1
50–59	3.11 (1.65–5.86)	<0.001	2.34 (1.19–4.60)	0.01	5.20 (6.33–42.73)	0.1	3.54 (0.41–30.3)	0.3
60–69	1.49 (0.76–2.91)	0.3	1.40 (0.71–2.76)	0.3	4.68 (0.53–41.07)	0.2	3.98 (0.45–34.31)	0.2
>70	Ref		Ref		Ref.		Ref	
**Higher education**								
Yes	1.04 (0.82–1.32)	0.8			0.79 (0.53–1.17)	0.2		
No	Ref				Ref			
**Married**								
Yes	Ref		Ref		Ref			
No	1.59 (1.24–2.02)	<0.001	1.27 (0.93–1.73)	0.1	1.32 (0.90–1.95)			
**Place of residence**								
Rural area	0.82 (0.55–1.22)	0.3			0.79 (0.42–1.50)	0.5		
City of <20,000 residents	0.88 (0.54–1.42)	0.6			0.84 (0.38–1.83)	0.7		
City of 20,000–99,999 residents	1.14 (0.74–1.75)	0.6			1.13 (0.58–2.23)	0.7		
City of 100,000–499,999 residents	1.03 (0.66–1.61)	0.9			1.16 (0.57–2.33)	0.7		
City of ≥500,000 residents	Ref				Ref.			
**Having children under 18 years**								
Yes	1.86 (1.43–2.43)	<0.001	1.21 (0.86–1.72)	0.3	1.63 (1.09–2.45)	0.02	1.41 (0.91–2.19)	0.1
No	Ref		Ref		Ref.		Ref.	
**Occupational status**								
Active (employed or self-employed)	2.83 (2.17–3.68)	<0.001	1.63 (1.19–2.24)	0.002	1.73 (1.08–2.77)	0.02	1.44 (0.86–2.42)	0.2
Passive (unemployed, student, or pensioner)	Ref		Ref		Ref.		Ref.	
**Self-declared household financial status**								
Good	1.16 (0.80–1.67)	0.4			0.64 (0.35–1.16)	0.1		
Moderate	1.14 (0.78–1.67)	0.5			0.71 (0.38–1.31)	0.3		
Bad	Ref				Ref.			

**Table A6:** Factors associated with the public perception of EDs (n = 1121).

Variable	Energy Drinks Are Harmful to Health				Support a Ban on the Sale of Energy Drinks to Minors			
	Bivariable Logistic Regression		Multivariable Logistic Regression		Bivariable Logistic Regression		Multivariable Logistic Regression	
	OR (95%CI)	*p*	aOR (95%CI)	*p*	OR (95%CI)	*p*	aOR (95%CI)	*p*
**Gender**								
Female	1.47 (1.08–2.01)	0.02	1.50 (1.09–2.06)	0.01	1.58 (1.11–2.24)	0.01	1.68 (1.16–2.41)	0.005
Male	Ref.		Ref.		Ref.		Ref.	
**Age [years]**								
18–29	0.74 (0.39–1.39)	0.3			Ref.		Ref.	
30–39	0.97 (0.52–1.80)	0.9			1.42 (0.84–2.40)	0.2	1.41 (0.81–2.45)	0.2
40–49	0.79 (0.43–1.45)	0.4			2.04 (1.17–3.56)	0.01	2.27 (1.25–4.11)	0.007
50–59	1.20 (0.63–2.28)	0.6			2.89 (1.56–5.33)	<0.001	3.11 (1.62–6.00)	<0.001
60–69	0.87 (0.47–1.62)	0.7			2.08 (1.17–3.71)	0.01	2.23 (1.19–4.16)	0.01
>70	Ref.				3.64 (1.62–8.20)	0.002	4.03 (1.74–9.34)	0.001
**Higher education**								
Yes	1.58 (1.14–2.18)	0.006	1.44 (1.03–2.00)	0.03	1.58 (1.10–2.27)	0.01	1.35 (0.92–1.97)	0.1
No	Ref.		Ref.		Ref.		Ref.	
**Married**								
Yes	1.39 (1.01–1.89)	0.04	1.36 (0.99–1.87)	0.06	1.68 (1.18–2.39)	0.04	1.26 (0.85–1.85)	0.2
No	Ref.		Ref.		Ref.		Ref.	
**Place of residence**								
Rural area	0.90 (0.53–1.53)	0.7			0.69 (0.37–1.28)	0.2		
City of < 20,000 residents	0.85 (0.45–1.61)	0.6			0.97 (0.45–2.10)	0.9		
City of 20,000–99,999 residents	0.93 (0.52–1.66)	0.8			0.72 (0.37–1.40)	0.3		
City of 100,000–499,999 residents	0.78 (0.44–1.41)	0.4			0.78 (0.39–1.57)	0.5		
City of ≥500,000 residents	Ref.				Ref.			
**Having children under 18 years**								
Yes	1.07 (0.75–1.52)	0.7			1.31 (0.87–1.98)	0.2		
No	Ref.				Ref.			
**Occupational status**								
Active (employed or self-employed)	1.14 (0.83–1.57)	0.4			1.15 (0.81–1.64)	0.4		
Passive (unemployed, student, or pensioner)	Ref.				Ref.			
**Self-declared household financial status**								
Good	1.20 (0.72–1.98)	0.5	1.09 (0.66–1.82)	0.7	2.26 (1.34–3.82)	0.002	2.35 (1.37–4.03)	0.002
Moderate	0.60 (0.37–0.98)	0.04	0.59 (0.36–0.96)	0.04	0.90 (0.55–1.47)	0.7	0.87 (0.53–1.43)	0.6
Bad	Ref.		Ref.		Ref.		Ref.	
